# Pulmonary adverse events associated with hypertension in non-small cell lung cancer patients receiving PD-1/PD-L1 inhibitors

**DOI:** 10.3389/fphar.2022.944342

**Published:** 2022-08-30

**Authors:** Jianing Chen, Yaokai Wen, Xiangling Chu, Yuzhi Liu, Chunxia Su

**Affiliations:** ^1^ School of Medicine, Tongji University, Shanghai, China; ^2^ Department of Medical Oncology, Shanghai Pulmonary Hospital & Thoracic Cancer Institute, Tongji University, School of Medicine, Shanghai, China; ^3^ Department of Oncology, Shanghai East Hospital, Tongji University, School of Medicine, Shanghai, China

**Keywords:** pharmacovigilance, immune checkpoint inhibitor, hypertension, NSCLC, FAERS

## Abstract

**Introduction:** Non-small cell lung cancer patients have gained therapeutic benefits from immune checkpoint inhibitors, although immune-related adverse events (irAEs) could be inevitable. Whether irAEs are associated with chronic diseases is still unclear, our study aims to clarify the distinct adverse events in NSCLC patients with concomitant hypertension.

**Methods:** Adverse event cases were searched and collected in the Food and Drug Administration (FDA) Adverse Event Reporting System (FAERS) database from January 2015 to December 2021. We performed disproportionality analysis to detect safety signals by calculating reporting odds ratios (ROR) and corresponding 95% confidence intervals (95% CIs), information component (IC), and the lower bound of the information component 95% credibility interval (IC_025_).

**Results:** Among 17,163 NSCLC patients under treatment with single-agent anti-programmed death-1/programmed death ligand-1 (PD-1/PD-L1) inhibitor (nivolumab, pembrolizumab, cemiplimab, durvalumab, atezolizumab, and avelumab), 497 patients had hypertension while 16,666 patients had no hypertension. 4,283 pulmonary AEs were reported, including 166 patients with hypertension and 4,117 patients without hypertension. Compared with patients without hypertension, patients with hypertension were positively associated with increased reporting of interstitial lung disease (ROR = 3.62, 95%CI 2.68–4.89, IC = 1.54, IC_025_ = 0.57) among patients receiving anti-PD-1 treatment. The median duration of onset from the time of initiation of anti-PD-1 administration was 28 days (IQR, 12.00–84.25).

**Conclusion:** Our pharmacovigilance analysis showed the profile of pulmonary toxicities in NSCLC patients with hypertension caused by anti-PD-1/PD-L1 inhibitors. Interstitial lung disease was the statistically significant reporting adverse event in patients with hypertension receiving anti-PD-1 treatment.

## Introduction

Immune checkpoint inhibitors (ICIs) that target the programmed death 1 receptor (PD-1) and programmed death-ligand 1 (PD-L1) have brought a durable long-term survival response to patients with malignant tumors. Nivolumab, pembrolizumab, cemiplimab, durvalumab, atezolizumab, and avelumab have been approved for non-small cell lung cancer (NSCLC). These approvals accelerated prescribing of these drugs in routine oncological practices. However, anti-tumor treatments also generate a series of unique dysimmune toxicities, which are termed as immune-related adverse events (irAEs) (Nishino et al., 2015; Tirumani et al., 2015; Michot et al., 2016). ICI-induced toxicities can cause suspension of the anti-tumor treatment, and some severe irAEs would impair life quality, even leading to death (Combs Scott and Pennell, 2017; Wang et al., 2018). Theoretically, irAEs can involve all organs and tissues (Champiat et al., 2016; Weber et al., 2017; Postow et al., 2018). Skin (Minkis et al., 2013; Abdel-Rahman et al., 2015), gastrointestinal tract (Di Giacomo et al., 2009; Gentile et al., 2013; Cheng et al., 2015), endocrine glands (Ryder et al., 2014; Albarel et al., 2015; Gaudy et al., 2015), and pulmonary system (Berthod et al., 2012; Barjaktarevic et al., 2013) are the most affected organs. The effective predictive biomarkers of irAEs are required to identify the risk for patients receiving anti-PD-1/PD-L1 administration. Patients with specific physical conditions are often at a high risk of irAEs. Therefore, before receiving immunotherapy, doctors need to carefully ask patients about their physical status. Patients with autoimmune disease (Kyi et al., 2014; Pedersen et al., 2014) and chronic infection (Sharma et al., 2013) are mentioned with a high risk of developing irAEs. Recently, biomarkers to predict irAEs have been reported, such as sex (Valpione et al., 2018), cytokines (Tarhini et al., 2015), autoantibodies (Duarte et al., 2018; Cortellini et al., 2019), TMB (Bomze et al., 2019), gut microbiome (Chaput et al., 2017), and multi-omics (Jing et al., 2020). However, the identification of candidate risk factors that prelude to irAEs is still a realm of highly unmet need.

Chronic conditions often lead to higher morbidity and mortality of malignant tumors. Aged patients with NSCLC are often associated with comorbidities, such as COPD, diabetes mellitus, hyperlipidemia, and hypertension. Hypertension, as a clinical factor, is the most frequently reported comorbidity in patients with malignancy, which has a reported prevalence of 38% (Piccirillo et al., 2004; Mouhayar and Salahudeen, 2011). Besides, hypertension is emerging as one of the most common side effects in NSCLC patients receiving immunotherapy (Garon et al., 2019). Its incidence increases significantly when combined with angiogenesis inhibitors including the anti-vascular endothelial growth factor (VEGF) monoclonal antibody bevacizumab (Jain et al., 2006; Ranpura et al., 2010; Syrigos et al., 2011) and certain small molecular inhibitors of tyrosine kinase (sunitinib, sorafenib, and pazopanib) (Riely and Miller, 2007).

The number of patients with lung cancer complicated with chronic diseases is very large, and the safety of immunotherapy in this population should not be ignored. However, patients with comorbidities such as uncontrolled hypertension are often excluded from oncological clinical trials. Whether patients with hypertension have a higher risk of irAEs is a lack of knowledge. Therefore, we aimed to investigate the association between irAEs and hypertension. Herein, we investigated the characteristics and risk factors of pulmonary ICI-related AEs through the FAERS database. Numerous researches suggested that the use of angiogenesis inhibitors can increase the risk of hypertension in cancer patients (Wu et al., 2008; Ranpura et al., 2010). In order to exclude the interference of other drug factors, our study only included reports of pulmonary adverse reactions after receiving single-agent immunotherapy.

## Methods

### Data Source and study design

Adverse event reports are available on FAERS database which is submitted by healthcare professionals, consumers, and manufacturers. The FAERS database contains demographic information, drug information, patient outcomes, and preferred terms (PTs) coded for the adverse events. These PTs are categorized into their primary system organ classes (SOCs) in the MedDRA and SOCs are equivalent to systematic classification in other medical terms. Our study was designed as a retrospective pharmacovigilance study. 14,072,154 FAERS records from January 2015 to December 2021 were included. According to the FDA’s recommendation, duplicate reports were removed by case number in this study, with only the most recent case version adopted. After extraction and de-duplication of case reports, there were 112,764 unique reports for patients who used anti-PD-1 (nivolumab, pembrolizumab, and cemiplimab) or anti-PD-L1 (durvalumab, atezolizumab, avelumab), then we excluded adverse events caused by combined therapies, only 63,055 cases receiving monotherapy included. 17,163 cases of non-small cell lung cancer (lung adenocarcinoma, lung squamous cell carcinoma/squamous cell carcinoma of lung, adenosquamous cell lung cancer, large cell lung cancer, sarcoid carcinoma, and not specified type of NSCLC) were finally included in our study, including 4,283 respiratory, thoracic and mediastinal AE reports. Severe adverse events were defined as death, life-threatening, disability, hospitalization, required for intervention, or any other outcomes.

### Statistical Analysis

Disproportionality analysis was applied to measure safety signals for patients who used anti-PD-1/PD-L1 therapy with hypertension under study (Almenoff et al., 2007). We calculated reporting odds ratios (ROR), 95% confidence intervals (95% CIs) and the lower bound of a two-sided 95% interval of information component (IC_025_) to detect potential associations between hypertension and irAEs (Bate et al., 1998; Bate et al., 2002; Bate and Evans, 2009). The calculation formulas for ROR and 95% CI were as follows: ROR = (a/c)/(b/d), 95% CI = e^ln(ROR) ± 1.96SQRT(1/a + 1/b + 1/c + 1/d)^. a = Number of patients with hypertension who received anti-PD1/PD-L1 therapy and developed the target irAEs. b = No. of hypertensive patients receiving anti-PD1/PD-L1 therapy with other adverse effects. c = No. of patients without hypertension who received anti-PD1/PD-L1 therapy and developed the target irAEs. d = No. of patients without hypertension receiving anti-PD1/PD-L1 therapy with other adverse effects. The safety signal was considered to be statistically significant when the ROR was greater than 1.0, IC more than zero and IC_025_ > 0. We also calculated the time-to onset of adverse events. The formula of the time-to-onset of events was as follows: Time-to-onset = Event onset date–Therapy start date. The median and interquartile ranges (IQR) were also calculated to show the time to onset.

RStudio (version 4.1.1; Boston, MA, United States) was used for all statistical analyses and for generating graphs in our study.

## Result

### Descriptive Analysis

**FIGURE 1:**
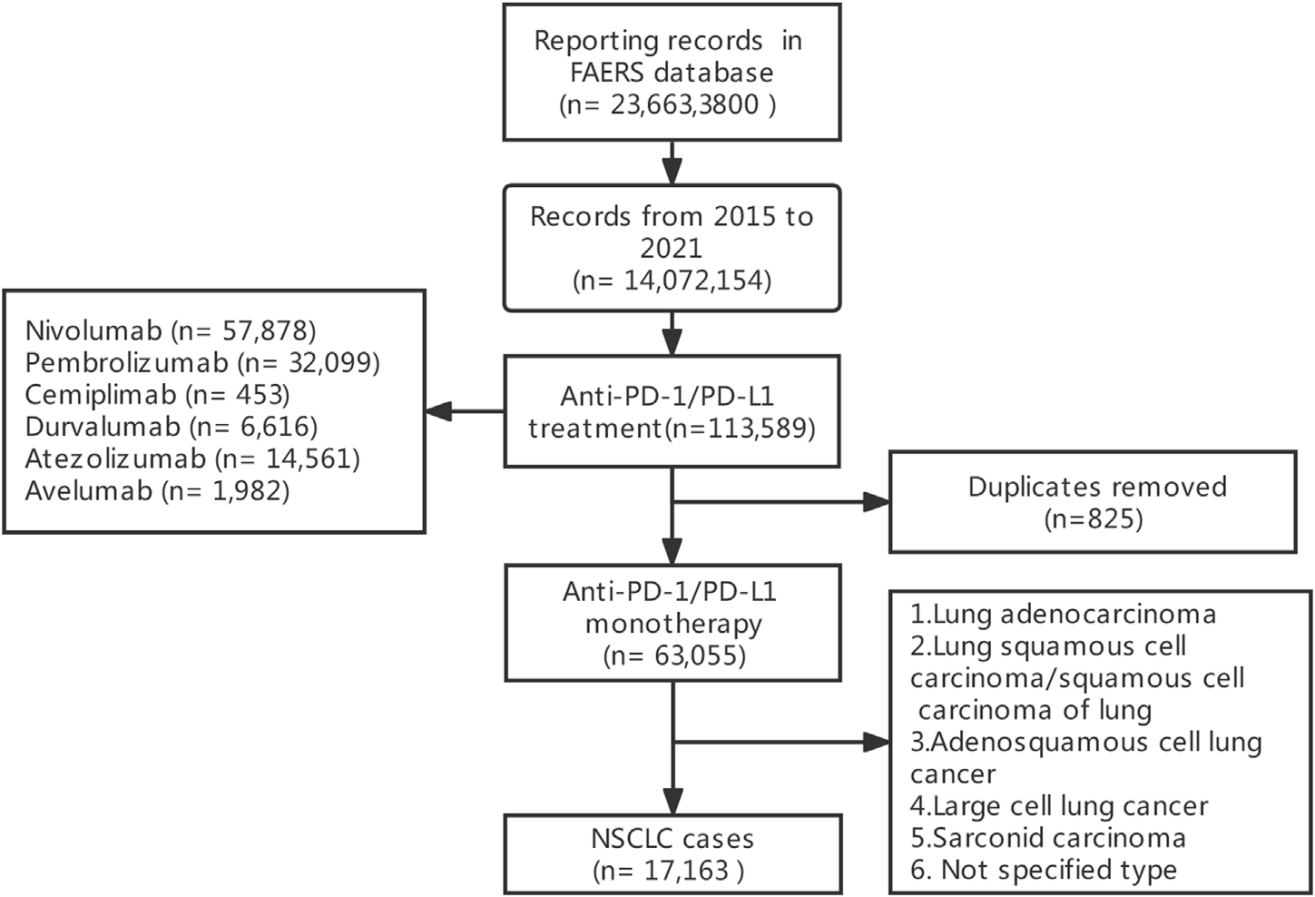
Flowchart of screening and inclusion of adverse reactions.

From 2015 to 2021, a total of 17,163 records were extracted (Figure 1), 497 patients were also diagnosed with hypertension 16,666 patients were diagnosed without hypertension. 4,283 (24.95%) were reported as respiratory thoracic and mediastinal AEs after using ICI regimes. Among them, 166 NSCLC patients were also diagnosed with hypertension. All demographic and clinical characteristics of patients were presented in Table 1. In the hypertensive and non-hypertensive groups, the proportion of males was higher than that of females. In the hypertensive group, the proportion of men (80.12%) was higher than that (67.09%) of the non-hypertensive group. In addition, compared to those aged younger than 65 years, higher percentage of patients older than 65 years in both cohorts (74.7%, 52.5%). Due to the severity of pulmonary irAEs, death was the most frequent report. Death (*n* = 76) was the most common outcome in hypertension cohort. Furthermore, death accounted for a larger proportion in hypertensive patients than that in non-hypertensive patients.

**TABLE 1:** Clinical characteristics of NSCLC patients with ICIs induced pulmonary toxicity, N (%).

	Hypertension (*n* = 166)	Without hypertension (*n* = 4,117)
Gender		
Male	133 (80.12)	2,762 (67.09)
Female	32 (19.28)	1,083 (26.31)
Missing	1 (0.60)	272 (6.61)
Age		
<65	34 (20.48)	1,134 (27.54)
≥65	124 (74.70)	2,176 (52.85)
Missing	8 (4.82)	807 (19.60)
Reporting year		
2015	16 (9.64)	164 (3.98)
2016	5 (3.01)	561 (13.63)
2017	38 (22.89)	728 (17.68)
2018	52 (31.33)	608 (14.77)
2019	35 (21.08)	506 (12.29)
2020	7 (4.22)	255 (6.19)
2021	3 (1.81)	156 (3.79)
Anti-PD-1		
Nivolumab	22 (13.25)	1784 (43.33)
Pembrolizumab	92 (55.42)	1,398 (33.96)
Cemiplimab	1 (0.60)	4 (0.10)
Anti-PD-L1		
Atezolizumab	20 (12.05)	325 (7.89)
Durvalumab	30 (18.07)	600 (14.57)
Avelumab	1 (0.60)	6 (0.15)
Outcome		
Death	76 (45.78)	1,437 (34.9)
Life-threatening	9 (5.42)	199 (4.83)
Hospitalization	68 (40.96)	1,270 (30.85)
Disability	2 (1.20)	19 (0.46)
Other serious	11 (6.63)	1,032 (25.07)
Non-Serious	0 (0)	160 (3.89)

### The number of adverse events for each drug

The distribution of SOCs for NSCLC patients was shown in Table 2. In total, general disorders (*n* = 4,493) and pulmonary disorders (*n* = 4,283) had the largest number of AEs. For patients receiving nivolumab, cemiplimab, or atezolizumab, the main irAEs were general disorders. For patients taking pembrolizumab, durvalumab or avelumab, the number of pulmonary disorders was the largest.

**TABLE 2:** System Organ Classes (SOCs) for adverse events of PD-1/PD-L1 inhibitors, N (%).

SOCs	Total	Nivolumab	Pembrolizumab	Cemiplimab	Durvalumab	Atezolizumab	Avelumab
General disorders and administration site conditions	4,493 (0.13)	2,268 (13.86)	1,325 (0.11)	6 (0.10)	464 (0.12)	424 (0.15)	6 (0.10)
Respiratory, thoracic and mediastinal disorders	4,283 (0.12)	1806 (11.03)	1,490 (0.12)	5 (0.08)	630 (0.17)	345 (0.12)	7 (0.12)
Neoplasms benign, malignant and unspecified	3,827 (0.11)	1730 (10.57)	1,404 (0.11)	0 (0)	558 (0.15)	135 (0.05)	0 (0)
Gastrointestinal disorders	2,578 (0.07)	1,286 (7.86)	917 (0.07)	5 (0.08)	160 (0.04)	202 (0.07)	8 (0.13)
Infections and infestations	2,456 (0.07)	1,148 (7.01)	828 (0.07)	7 (0.12)	237 (0.06)	231 (0.08)	5 (0.08)
Nervous system disorders	1953 (0.05)	926 (5.66)	677 (0.05)	3 (0.05)	144 (0.04)	199 (0.07)	4 (0.07)
Investigations	1823 (0.05)	810 (4.95)	665 (0.05)	3 (0.05)	167 (0.04)	173 (0.06)	5 (0.08)
Injury, poisoning and procedural complications	1904 (0.05)	786 (4.80)	457 (0.04)	1 (0.02)	578 (0.15)	79 (0.03)	3 (0.05)
Musculoskeletal and connective tissue disorders	1,512 (0.04)	785 (4.80)	503 (0.04)	1 (0.02)	119 (0.03)	102 (0.04)	2 (0.03)
Skin and subcutaneous tissue disorders	1,650 (0.05)	746 (4.56)	666 (0.05)	3 (0.05)	108 (0.03)	125 (0.04)	2 (0.03)
Cardiac disorders	1,257 (0.04)	589 (3.60)	422 (0.03)	4 (0.07)	121 (0.03)	119 (0.04)	2 (0.03)
Metabolism and nutrition disorders	1,284 (0.04)	582 (3.56)	464 (0.04)	5 (0.08)	69 (0.02)	159 (0.06)	5 (0.08)
Blood and lymphatic system disorders	1,071 (0.03)	518 (3.16)	374 (0.03)	5 (0.08)	70 (0.02)	103 (0.04)	1 (0.02)
Endocrine disorders	1,107 (0.03)	469 (2.87)	477 (0.04)	2 (0.03)	89 (0.02)	67 (0.02)	3 (0.05)
Hepatobiliary disorders	1,253 (0.04)	434 (2.65)	584 (0.05)	8 (0.14)	95 (0.02)	131 (0.05)	1 (0.02)
Renal and urinary disorders	912 (0.03)	371 (2.27)	398 (0.03)	0 (0)	41 (0.01)	102 (0.04)	0 (0)
Vascular disorders	616 (0.02)	296 (1.81)	208 (0.02)	0 (0)	58 (0.02)	53 (0.02)	1 (0.02)
Surgical and medical procedures	325 (0.01)	272 (1.66)	43 (<0.01)	0 (0)	7 (<0.01)	3 (<0.01)	0 (0)
Psychiatric disorders	435 (0.01)	186 (1.14)	170 (0.01)	1 (0.02)	34 (0.01)	44 (0.02)	0 (0)
Eye disorders	349 (0.01)	175 (1.07)	129 (0.01)	0 (0)	20 (0.01)	25 (0.01)	0 (0)
Immune system disorders	333 (0.01)	90 (0.55)	182 (0.01)	0 (0)	21 (0.01)	37 (0.01)	3 (0.05)
Ear and labyrinth disorders	86 (<0.01)	49 (0.30)	15 (<0.01)	0 (0)	11 (<0.01)	10 (<0.01)	1 (0.02)
Reproductive system and breast disorders	53 (<0.01)	23 (0.14)	17 (<0.01)	0 (0)	6 (<0.01)	7 (<0.01)	0 (0)
Product issues	21 (<0.01)	9 (0.05)	9 (<0.01)	0 (0)	1 (<0.01)	1 (<0.01)	1 (0.02)
Social circumstances	19 (<0.01)	7 (0.04)	8 (<0.01)	0 (0)	4 (<0.01)	0 (0)	0 (0)
Congenital, familial and genetic disorders	23 (<0.01)	6 (0.04)	13 (<0.01)	0 (0)	3 (<0.01)	1 (<0.01)	0 (0)

### The spectrum of pulmonary irAEs differed in PD-1 inhibitors.

**FIGURE 2:**
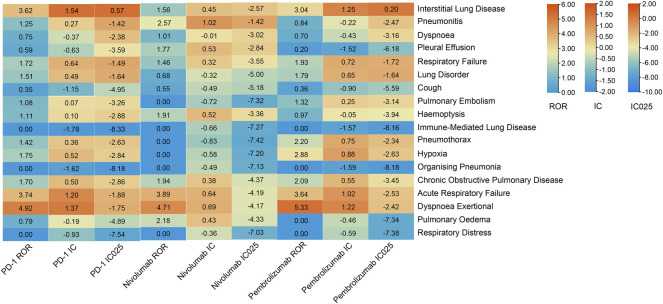
Safety signals of anti-PD-1 treatment in the NSCLC group with hypertension. ROR, reporting odds ratios; IC, information component; IC025, the lower limit of the 95% confidence interval of IC. ROR was greater than 1.0, the lower limit of 95% CI was above 1.0, IC more than zero and IC025 > 0. The value of each column is represented by a different color, the more orange the color, the larger the value. A signal is defined as ROR >1.0, IC > 0, and IC025 > 0.

**FIGURE 3:**
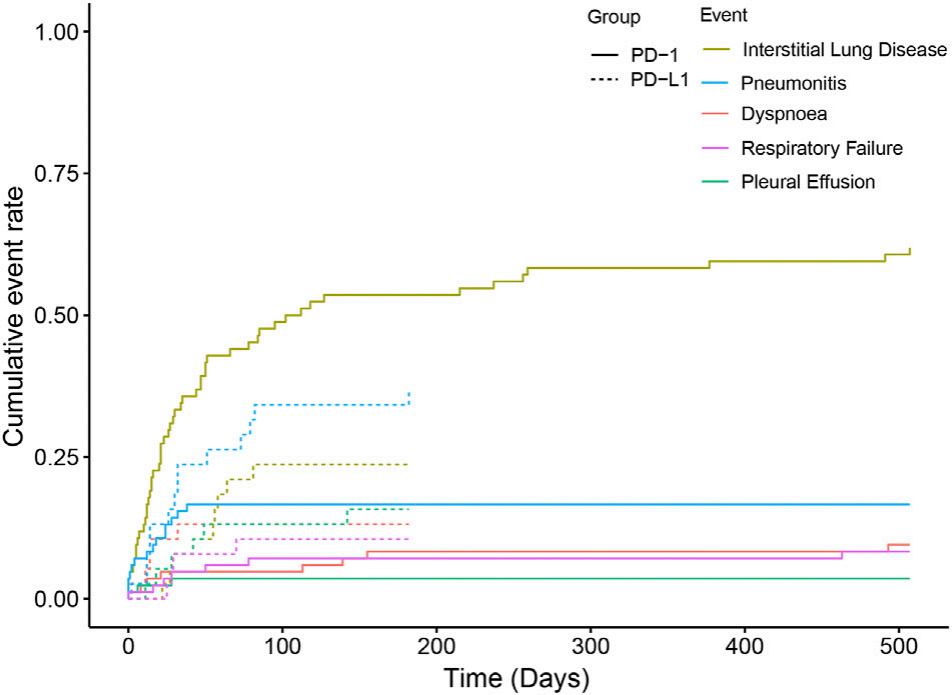
Time from initiation of ICI administration to onset of pulmonary adverse events.

The pulmonary signal spectrum of different anti-PD-1 therapies was shown in Figure 2 and Supplemetary Table S1. Cumulative event rates of irAEs since the initiation of ICI were shown in Figure 3. According to ROR and Bayesian confidence propagation neural network (BCPNN) algorithm, interstitial lung disease (ROR = 3.62, 95%CI 2.68–4.89, IC = 1.54, IC_025_ = 0.57) with median time-to-onset of 28 (12.00–84.25) days (Supplemetary Table S5), was the only one statistically positively associated with hypertension in patients receiving PD-1 inhibitors. Pneumonitis (ROR = 2.57, 95%CI 1.18–5.63, IC = 1.02, IC_025_ = −1.42) was not significantly correlated with hypertension in NSCLC patients receiving nivolumab. Interstitial lung disease (ROR = 3.04, 95%CI 2.19–4.23, IC = 1.25, IC_025_ = 0.20) was the mostly reported among the statistically significant reported adverse event in pembrolizumab subgroup. 54 patients with hypertension developed interstitial lung disease, with a disease severity rate of 100%, a mortality rate of 63%, and a hospitalization rate of 83%. Besides, we performed the disproportionality analysis of NSCLC patients without hypertension receiving anti-PD-1 treatment. The results demonstrated that no statistically significant signal was detected in the group without hypertension (Supplemetary Table S3,S4).

### The spectrum of pulmonary irAEs differed in PD-L1 inhibitors.

**FIGURE 4:**
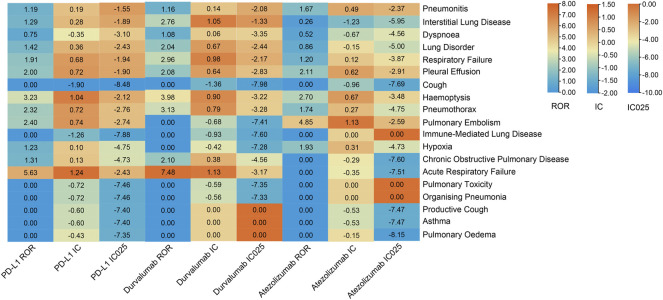
Safety signals of anti-PD-L1 therapy in the NSCLC group with hypertension. ROR, reporting odds ratios; IC, information component; IC025, the lower limit of the 95% confidence interval of IC. The value of each column is represented by a different color, the more orange the color, the larger the value. A signal is defined as ROR >1.0, IC > 0, and IC025 > 0.

The safety signal spectrum of different anti-PD-L1 treatments was presented in Figure 4 and Supplemetary Table S2. Using the ROR algorithm, haemoptysis (ROR 3.23, 95%CI 1.12–9.31) and acute respiratory failure (ROR 5.63, 95%CI 1.57–20.17) were mostly reported among the statistically significant reported adverse events in patients receiving PD-L1 inhibitors. However, when we used the Bayesian algorithm to estimate drug safety signals, neither of these achieved statistical significance (IC = 0.19, IC_025_ = −1.55; IC = 0.28, IC_025_ = −1.89). The median (IQR) time from therapy start to the onset of interstitial lung disease, pneumonitis, dyspnoea, pleural effusion and respiratory failure were 55 (29.00–58.00) days, 31 (14.00–67.50) days, 14 (11.00–14.00) days, 35 (20.50–47.25) days, 28.5 (27.25–39.25) days (Figure 3; Supplemetary Table S5).

## Discussion

Hypertension is one of the common chronic degenerative diseases, that involves remodeling and inflammation of arterial walls, and has an intricate relationship with cancer. Both of them share some same risk factors including smoking, diabetes mellitus, and physical inactivity (Battistoni et al., 2015; Ameri et al., 2018). Adjunctive therapies concurrently administered with antineoplastic agents can promote the development of hypertension or worsen previously controlled hypertension (Tonia et al., 2012; Cohen et al., 2019). Meanwhile, high blood pressure increases the risk of cancer development (Sanfilippo et al., 2014; Seretis et al., 2019). Dyer et al. (1977) firstly pointed out that hypertension might be a risk factor for cancer mortality, which was confirmed by other studies (Stocks et al., 2012; Berger et al., 2016; Harding et al., 2016) that hypertension could accelerate the biological process of aging which favors carcinogenesis. The metabolic disorders of hypertension increase oxidative stress and result in an irreversible proinflammatory state that reduces intracellular antioxidant capacity and predisposes it to malignant transformation (Federico et al., 2007). As hypertension is the most prevalent comorbidity in patients diagnosed with cancer (Piccirillo et al., 2004), patients with lung cancer coexisting with hypertension do not affect anti-tumor responses, nor does it affect the survival time (Yan et al., 2018). Common antihypertensive drugs, such as renin–angiotensin system inhibitors (RASi), angiotensin-converting enzyme inhibitors (ACEI), angiotensin II receptor blockers (ARBs) and direct renin inhibitors have no impact on clinical outcomes with anti-PD1/PD-L1 inhibitors (Bangalore et al., 2011; Cui et al., 2019).

Immunotherapeutic agents that target immune checkpoint pathways have shown great promise. Despite extensive research efforts, few biomarkers had a high accuracy and ubiquity to predict irAEs. Patients often receive additional concomitant therapies, which bring a lot of confounding factors to the risk of irAEs to immunotherapy. Concomitant medications in the treatment of malignant tumors have different effects on response to immunotherapy (Arbour et al., 2018; Fuca et al., 2019). Some reports found that antibiotics had detrimental efficacy and toxicity effects on ICIs. In fact, compare with patients without receiving extra agents, patients receiving baseline concomitant medication had worse outcomes (Cortellini et al., 2020). We have already known that antibiotics could increase the risk of irAE by changing the gut microbiome (Pinato et al., 2019). Not only is hypertension a common adverse reaction, frequently reported in clinical trials, but also a common comorbidity in patients with non-small cell lung cancer. However, the safety of antineoplastic therapy in these patients with hypertension has been rarely reported. As a common adverse reaction, the incidence of arterial hypertension is associated with the clinical outcome of antiangiogenetic-targeted treatment modalities in patients with tumors. (Scartozzi et al., 2009). As there were growing reports on the relationship between the occurrence of irAEs and tumor response, the anti-tumor treatments of ICI were associated with a reduced incidence of irAEs (Teraoka et al., 2017; Sato et al., 2018). We speculated that there is a potential link between high blood pressure and adverse reactions.

Although the causative pathogenic mechanism of hypertension-associated irAEs was poorly understood, studies have suggested that activation or reactivation of tissue-resident autoreactive T cells is thought to be a dominant prime factor in the development of irAEs (June et al., 2017; Dougan et al., 2021). Shared antigens between the specified organs and vessels could lead to *de novo* T cell activation and precipitate unwanted effects. High blood pressure caused endothelial dysfunction and vascular oxidative stress, leading to vasoconstriction. Neoantigens generated and then T cells were activated by binding specific antigens presented in major histocompatibility complex molecules on specific antigen-presenting cells, thereby activating of the adaptive immune system (Vinh et al., 2010). Activated T cells infiltrated blood vessels and produced cytokines, which promoted endothelial dysfunction and low-grade chronic inflammation (Idris-Khodja et al., 2014). Beyond increased perivascular immune cells accumulation and intravascular infiltration, circulating levels of certain cytokines and chemokines are abnormally elevated. Multiple chemokines recruited and stimulated the infiltration of T cells and monocytes and macrophages during hypertension (Guzik et al., 2007; Moore et al., 2015; Mikolajczyk et al., 2016). Besides, elevated circulatory levels of cytokines, C-reactive proteins, and immunoglobulins in patients with hypertension have also been reported. furthermore, autoreactive antibodies to vascular wall antigens have been detected (Martinez Amenos et al., 1985; Blake et al., 2003; Alexander et al., 2019). Recent investigations demonstrated that circulating antibody levels are elevated in both essential and pregnancy-related hypertension (Dib et al., 2012; Chan et al., 2014). Together, these studies indicated that T cells could be activated when self-peptides are presented through epitopes spread by antigen-presenting cells. Pre-existing autoreactive T cells have already existed and be kept in check through immune checkpoint molecules. When receiving immune checkpoint inhibitors, immune cells were over-activated, resulting in a low-level inflammatory response in tumor patients being amplified, further leading to immune-related adverse reactions.

To our knowledge, irAEs after receiving PD-1/PD-L1 inhibitors have never been reported in the context of cancer patients under chronic diseases. According to real-world data, we found a high reporting frequency of respiratory AEs associated with PD-1/PD-L1 inhibitors. Meanwhile, every PD-1/PD-L1 inhibitor has respective profiles of toxicities. Our study showed statistical evidence regarding the association between pulmonary irAEs and hypertension, which needs to be interpreted cautiously and further verified in pharmacology and clinical aspects. Beyond that, there may be some other potential mechanisms that could affect the safety of immunotherapies. Chronic diseases, particularly in aged patients, have an indirect causative effect on the occurrence of irAE. They need to pay attention to pulmonary adverse reactions during immunotherapy. Our study could help to recognize and manage irAEs in clinical practice. Further observational studies are required to establish the safety of ICIs in hypertensive patients.

We acknowledged several limitations in our study beyond its retrospective and observative nature, with reporting bias, missing data, and confounding bias on the FAERS database, specific grades of hypertension, and cancer outcomes. We would prospectively assess the physical condition of NSCLC patients and investigated interactions between hypertension and irAEs in our center to validate our results. In addition, we need to further analyze the clinical outcomes in NSCLC patients with hypertension.

## Conclusion

NSCLC patients with hypertension receiving PD-1/PD-L1 inhibitors have higher reporting odds of pulmonary adverse events. Clinicians should pay special attention to the occurrence of interstitial lung disease when using immunotherapy for these patients, and should intervene in time if lung disease occurs. Other adverse events such as pneumonitis and haemoptysis, which were highly reported without significance by the Bayesian IC algorithm, should not be ignored in clinical practice.

## Data Availability

The original contributions presented in the study are included in the article/Supplementary Material, further inquiries can be directed to the corresponding authors.
