# Supplementary material for: Pulmonary adverse events associated with hypertension in non-small cell lung cancer patients receiving PD-1/PD-L1 inhibitors
Source: Front Pharmacol. 2022 Aug 30;13:944342. doi: 10.3389/fphar.2022.944342 (PMC9468816; doi:10.3389/fphar.2022.944342)
Supplement: Supplementary file 1 [file DataSheet1.docx]

**Table S1** Disproportionality analysis of NSCLC patients with hypertension receiving anti-PD-1 treatment

| **Event** | **PD-1 inhibitors** | | | | **Nivolumab** | | | | **Pembrolizumab** | | | |
| --- | --- | --- | --- | --- | --- | --- | --- | --- | --- | --- | --- | --- |
|  | **N** | **ROR (95%CI)** | **IC** | **IC025** | **N** | **ROR (95%CI)** | **IC** | **IC025** | **N** | **ROR (95%CI)** | **IC** | **IC025** |
| Interstitial Lung Disease | 54 | 3.62(2.68-4.89) | 1.54 | 0.57 | 4 | 1.56(0.57-4.3) | 0.45 | -2.57 | 49 | **3.04(2.19-4.23)** | **1.25** | **0.20** |
| Pneumonitis | 15 | 1.25(0.74-2.11) | 0.27 | -1.42 | 7 | 2.57(1.18-5.63) | 1.02 | -1.42 | 8 | 0.84(0.41-1.72) | -0.22 | -2.47 |
| Dyspnoea | 10 | 0.75(0.4-1.41) | -0.37 | -2.38 | 4 | 1.01(0.37-2.77) | -0.01 | -3.02 | 5 | 0.7(0.28-1.72) | -0.43 | -3.16 |
| Pleural Effusion | 4 | 0.59(0.22-1.58) | -0.63 | -3.59 | 3 | 1.77(0.55-5.65) | 0.53 | -2.84 | 1 | 0.2(0.03-1.46) | -1.52 | -6.18 |
| Respiratory Failure | 9 | 1.72(0.88-3.39) | 0.64 | -1.49 | 2 | 1.46(0.36-6.02) | 0.32 | -3.55 | 7 | 1.93(0.88-4.23) | 0.72 | -1.72 |
| Lung Disorder | 9 | 1.51(0.77-2.97) | 0.49 | -1.64 | 1 | 0.68(0.09-4.91) | -0.32 | -5.00 | 8 | 1.79(0.86-3.71) | 0.65 | -1.64 |
| Cough | 2 | 0.35(0.09-1.4) | -1.15 | -4.95 | 1 | 0.55(0.08-3.99) | -0.49 | -5.18 | 1 | 0.36(0.05-2.61) | -0.90 | -5.59 |
| Pulmonary Embolism | 3 | 1.08(0.34-3.43) | 0.07 | -3.26 | 0 | 0.00 | -0.72 | -7.32 | 3 | 1.32(0.41-4.27) | 0.25 | -3.14 |
| Haemoptysis | 4 | 1.11(0.41-3.01) | 0.10 | -2.88 | 2 | 1.91(0.46-7.87) | 0.52 | -3.36 | 2 | 0.97(0.23-4.03) | -0.05 | -3.94 |
| Immune-Mediated Lung Disease | 0 | 0.00 | -1.78 | -8.33 | 0 | 0.00 | -0.66 | -7.27 | 0 | 0.00 | -1.57 | -8.16 |
| Pneumothorax | 4 | 1.42(0.52-3.88) | 0.36 | -2.63 | 0 | 0.00 | -0.83 | -7.42 | 4 | 2.2(0.78-6.18) | 0.75 | -2.34 |
| Hypoxia | 3 | 1.75(0.55-5.6) | 0.52 | -2.84 | 0 | 0.00 | -0.58 | -7.20 | 3 | 2.88(0.86-9.64) | 0.88 | -2.63 |
| Organising Pneumonia | 0 | 0.00 | -1.62 | -8.18 | 0 | 0.00 | -0.49 | -7.13 | 0 | 0.00 | -1.59 | -8.18 |
| Chronic Obstructive Pulmonary Disease | 3 | 1.7(0.53-5.43) | 0.50 | -2.86 | 1 | 1.94(0.26-14.22) | 0.38 | -4.37 | 2 | 2.09(0.49-8.93) | 0.55 | -3.45 |
| Acute Respiratory Failure | 4 | 3.74(1.33-10.5) | 1.20 | -1.88 | 1 | 3.89(0.52-29.13) | 0.64 | -4.19 | 3 | 3.64(1.07-12.4) | 1.02 | -2.53 |
| Dyspnoea Exertional | 4 | 4.92(1.73-13.98) | 1.37 | -1.75 | 1 | 4.71(0.62-35.59) | 0.69 | -4.17 | 3 | 5.33(1.51-18.84) | 1.22 | -2.42 |
| Pulmonary Oedema | 1 | 0.79(0.11-5.77) | -0.19 | -4.89 | 1 | 2.18(0.3-16.01) | 0.43 | -4.33 | 0 | 0.00 | -0.46 | -7.34 |
| Respiratory Distress | 0 | 0.00 | -0.93 | -7.54 | 0 | 0.00 | -0.36 | -7.03 | 0 | 0.00 | -0.59 | -7.38 |

**Significant signals are shown in bold italics.**

**Table S2** Disproportionality analysis of NSCLC patients with hypertension receiving anti-PD-L1 treatment.

| **Event** | **PD-L1 inhibitors** | |  |  | **Durvalumab** | |  |  | **Atezolizumab** | |  |  |
| --- | --- | --- | --- | --- | --- | --- | --- | --- | --- | --- | --- | --- |
|  | **N** | **ROR (95%CI)** | **IC** | **IC025** | **N** | **ROR (95%CI)** | **IC** | **IC025** | **N** | **ROR (95%CI)** | **IC** | **IC025** |
| Pneumonitis | 15 | 1.19(0.69-2.04) | 0.19 | -1.55 | 9 | 1.16(0.57-2.37) | 0.14 | -2.08 | 5 | 1.67(0.19-14.7) | 0.49 | -2.37 |
| Interstitial Lung Disease | 9 | 1.29(0.65-2.57) | 0.28 | -1.89 | 8 | 2.76(1.29-5.93) | 1.05 | -1.33 | 1 | 0.26(0.02-4.18) | -1.23 | -5.95 |
| Dyspnoea | 5 | 0.75(0.3-1.85) | -0.35 | -3.10 | 3 | 1.08(0.33-3.52) | 0.06 | -3.35 | 2 | 0.52(0.05-5.86) | -0.67 | -4.56 |
| Lung Disorder | 5 | 1.42(0.57-3.55) | 0.36 | -2.43 | 3 | 2.04(0.72-5.77) | 0.67 | -2.44 | 1 | 0.86(0.05-14.38) | -0.15 | -5.00 |
| Respiratory Failure | 6 | 1.91(0.82-4.46) | 0.68 | -1.94 | 4 | 2.96(1.03-8.49) | 0.98 | -2.17 | 2 | 1.2(0.1-13.69) | 0.12 | -3.87 |
| Pleural Effusion | 6 | 2(0.85-4.67) | 0.72 | -1.90 | 4 | 2.08(0.63-6.88) | 0.64 | -2.83 | 3 | 2.11(0.21-21.23) | 0.62 | -2.91 |
| Cough | 0 | 0.00 | -1.90 | -8.48 | 0 | 0 | -1.36 | -7.98 | 0 | 0 | -0.96 | -7.69 |
| Haemoptysis | 4 | 3.23(1.12-9.31) | 1.04 | -2.12 | 0 | 3.98(0.89-17.72) | 0.90 | -3.22 | 2 | 2.7(0.23-31.93) | 0.67 | -3.48 |
| Pneumothorax | 3 | 2.32(0.7-7.7) | 0.72 | -2.76 | 2 | 3.13(0.72-13.71) | 0.79 | -3.28 | 1 | 1.74(0.1-29.97) | 0.27 | -4.75 |
| Pulmonary Embolism | 3 | 2.4(0.72-7.99) | 0.74 | -2.74 | 2 | 0 | -0.68 | -7.41 | 3 | 4.85(0.46-50.83) | 1.13 | -2.59 |
| Immune-Mediated Lung Disease | 0 | 0.00 | -1.26 | -7.88 | 0 | 0 | -0.93 | -7.60 | 0 | 0 | 0.00 | 0.00 |
| Hypoxia | 1 | 1.23(0.16-9.3) | 0.10 | -4.75 | 1 | 0 | -0.42 | -7.28 | 1 | 1.93(0.11-33.57) | 0.31 | -4.73 |
| Chronic Obstructive Pulmonary Disease | 1 | 1.31(0.17-9.89) | 0.13 | -4.73 | 0 | 2.1(0.27-16.2) | 0.38 | -4.56 | 0 | 0 | -0.29 | -7.60 |
| Acute Respiratory Failure | 3 | 5.63(1.57-20.17) | 1.24 | -2.43 | 0 | 7.48(1.56-35.86) | 1.13 | -3.17 | 0 | 0 | -0.35 | -7.51 |
| Pulmonary Toxicity | 0 | 0.00 | -0.72 | -7.46 | 0 | 0 | -0.59 | -7.35 | 0 | 0 | 0.00 | 0.00 |
| Organising Pneumonia | 0 | 0.00 | -0.72 | -7.46 | 2 | 0 | -0.56 | -7.33 | 0 | 0 | 0.00 | 0.00 |
| Productive Cough | 0 | 0.00 | -0.60 | -7.40 | 0 | 0 | 0.00 | 0.00 | 0 | 0 | -0.53 | -7.47 |
| Asthma | 0 | 0.00 | -0.60 | -7.40 | 0 | 0 | 0.00 | 0.00 | 0 | 0 | -0.53 | -7.47 |
| Pulmonary Oedema | 0 | 0.00 | -0.43 | -7.35 | 0 | 0 | 0.00 | 0.00 | 0 | 0 | -0.15 | -8.15 |

**Table S3** Disproportionality analysis of NSCLC patients without hypertension receiving anti-PD-1 treatment

| **Event** | **PD-1 inhibitors** | | | | **Nivolumab** | | | | **Pembrolizumab** | | | |
| --- | --- | --- | --- | --- | --- | --- | --- | --- | --- | --- | --- | --- |
|  | **N** | **ROR (95%CI)** | **IC** | **IC025** | **N** | **ROR (95%CI)** | **IC** | **IC025** | **N** | **ROR (95%CI)** | **IC** | **IC025** |
| Interstitial Lung Disease | 658 | 0.28(0.2-0.37) | -0.08 | -0.42 | 231 | 0.64(0.23-1.76) | -0.01 | -0.61 | 427 | 0.33(0.24-0.46) | -0.10 | -0.51 |
| Pneumonitis | 473 | 0.8(0.47-1.36) | -0.01 | -0.42 | 254 | 0.39(0.18-0.85) | -0.03 | -0.59 | 217 | 1.19(0.58-2.45) | 0.00 | -0.60 |
| Dyspnoea | 515 | 1.33(0.71-2.52) | 0.01 | -0.39 | 352 | 0.99(0.36-2.72) | 0.00 | -0.48 | 162 | 1.43(0.58-3.52) | 0.01 | -0.70 |
| Pleural Effusion | 264 | 1.71(0.63-4.61) | 0.01 | -0.55 | 153 | 0.57(0.18-1.81) | -0.02 | -0.75 | 111 | 4.93(0.69-35.5) | 0.04 | -0.82 |
| Respiratory Failure | 206 | 0.58(0.3-1.14) | -0.03 | -0.66 | 122 | 0.68(0.17-2.81) | -0.02 | -0.84 | 84 | 0.52(0.24-1.13) | -0.07 | -1.04 |
| Lung Disorder | 234 | 0.66(0.34-1.3) | -0.02 | -0.61 | 130 | 1.47(0.2-10.67) | -0.01 | -0.80 | 104 | 0.56(0.27-1.16) | -0.06 | -0.93 |
| Cough | 222 | 2.88(0.71-11.64) | 0.02 | -0.59 | 159 | 1.81(0.25-13.08) | 0.00 | -0.72 | 63 | 2.78(0.38-20.1) | 0.02 | -1.12 |
| Pulmonary Embolism | 108 | 0.92(0.29-2.92) | -0.02 | -0.89 | 56 | 0.00 | -0.01 | -1.22 | 52 | 0.76(0.23-2.44) | -0.04 | -1.29 |
| Haemoptysis | 141 | 0.9(0.33-2.46) | -0.01 | -0.78 | 94 | 0.52(0.13-2.16) | -0.03 | -0.96 | 47 | 1.03(0.25-4.26) | -0.03 | -1.34 |
| Immune-Mediated Lung Disease | 96 | 0.00 | 0.02 | -0.91 | 50 | 0.00 | -0.01 | -1.30 | 46 | 0.00 | 0.03 | -1.31 |
| Pneumothorax | 110 | 0.7(0.26-1.92) | -0.03 | -0.89 | 68 | 0.00 | 0.00 | -1.11 | 42 | 0.46(0.16-1.28) | -0.10 | -1.47 |
| Hypoxia | 67 | 0.57(0.18-1.82) | -0.05 | -1.15 | 43 | 0.00 | -0.02 | -1.40 | 24 | 0.35(0.1-1.16) | -0.15 | -1.94 |
| Organising Pneumonia | 82 | 0.00 | 0.02 | -0.99 | 35 | 0.00 | -0.02 | -1.55 | 47 | 0.00 | 0.03 | -1.29 |
| Chronic Obstructive Pulmonary Disease | 69 | 0.59(0.18-1.88) | -0.04 | -1.13 | 46 | 0.52(0.07-3.78) | -0.04 | -1.38 | 22 | 0.48(0.11-2.05) | -0.12 | -1.99 |
| Acute Respiratory Failure | 42 | 0.27(0.1-0.75) | -0.12 | -1.49 | 23 | 0.26(0.03-1.93) | -0.10 | -1.96 | 19 | 0.27(0.08-0.93) | -0.20 | -2.19 |
| Dyspnoea Exertional | 32 | 0.2(0.07-0.58) | -0.17 | -1.72 | 19 | 0.21(0.03-1.6) | -0.12 | -2.15 | 13 | 0.19(0.05-0.66) | -0.30 | -2.65 |
| Pulmonary Oedema | 49 | 1.26(0.17-9.14) | -0.02 | -1.31 | 41 | 0.46(0.06-3.38) | -0.05 | -1.46 | 8 | 0.00 | -0.10 | -3.16 |
| Respiratory Distress | 35 | 0.00 | 0.00 | -1.54 | 24 | 0.00 | -0.04 | -1.88 | 11 | 0.00 | -0.06 | -2.71 |

**Table S4** Disproportionality analysis of NSCLC patients without hypertension receiving anti-PD-L1 therapy

| **Event** | **PD-L1 inhibitors** | |  |  | **Durvalumab** | |  |  | **Atezolizumab** | |  |  |
| --- | --- | --- | --- | --- | --- | --- | --- | --- | --- | --- | --- | --- |
|  | **N** | **ROR (95%CI)** | **IC** | **IC025** | **N** | **ROR (95%CI)** | **IC** | **IC025** | **N** | **ROR (95%CI)** | **IC** | **IC025** |
| Pneumonitis | 285 | 0.84(0.49-1.46) | -0.01 | -0.53 | 230 | 0.86(0.42-1.75) | -0.01 | -0.58 | 53 | 0.6(0.07-5.28) | -0.07 | -2.57 |
| Interstitial Lung Disease | 157 | 0.78(0.39-1.55) | -0.02 | -0.73 | 91 | 0.36(0.17-0.78) | -0.08 | -1.00 | 65 | 3.9(0.24-63.52) | 0.04 | -1.07 |
| Dyspnoea | 147 | 1.34(0.54-3.31) | 0.01 | -0.73 | 81 | 0.92(0.28-2.99) | -0.02 | -1.01 | 65 | 1.92(0.17-21.71) | 0.02 | -1.08 |
| Pleural Effusion | 68 | 0.5(0.21-1.17) | -0.08 | -1.15 | 43 | 0.48(0.15-1.59) | -0.08 | -1.43 | 25 | 0.47(0.05-4.76) | -0.13 | -1.87 |
| Respiratory Failure | 71 | 0.52(0.22-1.22) | -0.07 | -1.12 | 41 | 0.34(0.12-0.97) | -0.11 | -1.49 | 29 | 0.84(0.07-9.58) | -0.06 | -7.04 |
| Lung Disorder | 79 | 0.71(0.28-1.77) | -0.04 | -1.04 | 59 | 0.49(0.17-1.39) | -0.07 | -1.22 | 20 | 1.16(0.07-19.38) | -0.05 | -1.07 |
| Cough | 62 | 0.00 | 0.04 | -1.11 | 46 | 0.00 | 0.02 | -1.31 | 16 | 0.00 | 0.00 | 0.00 |
| Pulmonary Embolism | 28 | 0.42(0.13-1.38) | -0.12 | -1.79 | 17 | 0.00 | -0.03 | -2.19 | 11 | 0.21(0.02-2.16) | -0.34 | -2.85 |
| Haemoptysis | 28 | 0.31(0.11-0.89) | -0.17 | -1.82 | 15 | 0.25(0.06-1.12) | -0.20 | -2.43 | 13 | 0.37(0.03-4.39) | -0.20 | -1.08 |
| Pneumothora-x | 29 | 0.43(0.13-1.43) | -0.12 | -1.75 | 19 | 0.32(0.07-1.4) | -0.15 | -2.16 | 10 | 0.58(0.03-9.95) | -0.16 | -2.57 |
| Hypoxia | 18 | 0.81(0.11-6.11) | -0.08 | -2.16 | 9 | 0.00 | -0.09 | -3.00 | 9 | 0.52(0.03-9.02) | -0.19 | -1.08 |
| Chronic Obstructive Pulmonary Disease | 17 | 0.76(0.1-5.78) | -0.09 | -2.23 | 14 | 0.48(0.06-3.67) | -0.13 | -2.46 | 3 | 0.00 | -0.26 | 0.00 |
| Immune-Mediated Lung Disease | 31 | 0.00 | 0.02 | -1.60 | 26 | 0.00 | 0.00 | -1.76 | 0 | 0.00 | 0.00 | -1.08 |
| Productive Cough | 11 | 0.00 | -0.06 | -2.71 | 0 | 0.00 | 0.00 | 0.00 | 7 | 0.00 | -0.10 | -2.57 |
| Epistaxis | 5 | 0.00 | -0.17 | -3.92 | 0 | 0.00 | 0.00 | 0.00 | 2 | 0.00 | -0.35 | -1.08 |
| Acute Respiratory Failure | 12 | 0.18(0.05-0.64) | -0.33 | -2.75 | 8 | 0.13(0.03-0.64) | -0.37 | -3.29 | 4 | 0.00 | -0.19 | 0.00 |
| Pulmonary Haemorrhage | 6 | 0.00 | -0.14 | -3.61 | 0 | 0.00 | 0.00 | 0.00 | 2 | 0.00 | -0.35 | -5.66 |
| Pulmonary Toxicity | 14 | 0.00 | -0.03 | -2.41 | 14 | 0.00 | -0.05 | -2.41 | 0 | 0.00 | 0.00 | -7.04 |
| Asthma | 11 | 0.00 | -0.06 | -2.71 | 0 | 0.00 | 0.00 | 0.00 | 7 | 0.00 | -0.10 | 0.00 |

**Table S5** Time from ICI initiation to adverse events onset

| **Event** | **Time to onset, median [IQR]** | |
| --- | --- | --- |
|  | **PD-1** | **PD-L1** |
| Interstitial Lung Disease | 28(12.00-84.25) | 55(29.00-58.00) |
| Pneumonitis | 14(1.25-24.00) | 31(14.00-67.50) |
| Dyspnoea | 67(11.00-143.00) | 14(11.00-14.00) |
| Pleural Effusion | 6(3.00-17.00) | 35(20.50-47.25) |
| Respiratory Failure | 28(19.50-64.00) | 28.5(27.25-39.25) |
